# 

**Published:** 1848-04

**Authors:** 


					vol, mi.
APRIL, 1848.
No. I
THE
AMERICAN
IIL AID LIBRARY
OF
Denial fftxtnte.
"^.*3 A!1'."' * :WMW! WW
UB LIS HfD
QUARTERLY.
PUBLISHED UNDER THE AUSPICES QT THE
&merCcau Sboctetg of Bents* Sursron^
editors:
CHAPIN A; HARRIS, M. D., D. D. S.
AMOS WESTCOTT, M. D., D. D. S.
WILLIAM H. DWINELLE, D. D. S.
CORRESPONDENT FOR ENGLAND :
JAMES ROBINSON, D. D, S.,7 Gower-st. Bedford Square, London.
BALTIMORE:
ARMSTRONG 8t BERRY,
PHILADELPHIA: LINDSAY & BLAKISTON
JOHN W. WOODS, PRINTER.
I 111 M l' l|l . i _ l 1."' l
[j 9 5 OO per annum, payable in advance.
This Periodical weighs 9 ounces.

				

